# Implementation and comparison of two text mining methods with a standard pharmacovigilance method for signal detection of medication errors

**DOI:** 10.1186/s12911-020-1097-0

**Published:** 2020-05-24

**Authors:** Nadine Kadi Eskildsen, Robert Eriksson, Sten B. Christensen, Tamilla Stine Aghassipour, Mikael Juul Bygsø, Søren Brunak, Suzanne Lisbet Hansen

**Affiliations:** 1 Department of Safety Surveillance, Global Safety, Novo Nordisk A/S, Bagsværd, Denmark; 2 Disease Systems Biology Program, Novo Nordisk Foundation Center for Protein Research, University of Copenhagen, Copenhagen, Denmark; 3 Global Information & Analysis, Novo Nordisk A/S, Bagsværd, Denmark

**Keywords:** Signal detection, Pharmacovigilance, Individual case reports, Medication errors, Natural language processing, Text mining, Precision, Recall

## Abstract

**Background:**

Medication errors have been identified as the most common preventable cause of adverse events. The lack of granularity in medication error terminology has led pharmacovigilance experts to rely on information in individual case safety reports’ (ICSRs) codes and narratives for signal detection, which is both time consuming and labour intensive. Thus, there is a need for complementary methods for the detection of medication errors from ICSRs. The aim of this study is to evaluate the utility of two natural language processing text mining methods as complementary tools to the traditional approach followed by pharmacovigilance experts for medication error signal detection.

**Methods:**

The safety surveillance advisor (SSA) method, I2E text mining and University of Copenhagen Center for Protein Research (CPR) text mining, were evaluated for their ability to extract cases containing a type of medication error where patients *extracted insulin from a prefilled pen or cartridge by a syringe*. A total of 154,209 ICSRs were retrieved from Novo Nordisk’s safety database from January 1987 to February 2018. Each method was evaluated by recall (sensitivity) and precision (positive predictive value).

**Results:**

We manually annotated 2533 ICSRs to investigate whether these contained the sought medication error. All these ICSRs were then analysed using the three methods. The recall was 90.4, 88.1 and 78.5% for the CPR text mining, the SSA method and the I2E text mining, respectively. Precision was low for all three methods ranging from 3.4% for the SSA method to 1.9 and 1.6% for the CPR and I2E text mining methods, respectively.

**Conclusions:**

Text mining methods can, with advantage, be used for the detection of complex signals relying on information found in unstructured text (e.g., ICSR narratives) as standardised and both less labour-intensive and time-consuming methods compared to traditional pharmacovigilance methods. The employment of text mining in pharmacovigilance need not be limited to the surveillance of potential medication errors but can be used for the ongoing regulatory requests, e.g., obligations in risk management plans and may thus be utilised broadly for signal detection and ongoing surveillance activities.

## Background

A medication error is defined as the unintended failure in drug treatment occurring at any stage of the treatment process that results or can result in patient harm [1]. Medication errors have been identified as the most common preventable cause of adverse events and a significant source of economic burden for healthcare systems [2]. For European healthcare systems, for example, estimates of the yearly economic impact of medication errors range between 4.5 and 21.8 billion euros [2]. Furthermore, the reporting of medication errors has increased at a steady pace since 2005, which may be due to factors such as changes to the Medical Dictionary for Regulatory Activities (MedDRA®) terminology, increased awareness or a generally increased risk for medication errors as more medications with complex devices become available [3]. In an effort to prevent patient harm due to medication errors, various stakeholders such as the World Health Organization (WHO), the European Medicines Agency (EMA) and the Food and Drug Administration (FDA) have created guidelines and imposed legislations aiming at improving the reporting and detection of medication errors [3–7]. One of these mitigation efforts is the EU pharmacovigilance legislation from 2012. The legislation requires that all suspected serious and non-serious adverse reactions associated with medication errors must be reported by national competent authorities and marketing authorisation holders to the EudraVigilance system, which is the EMA’s database for safety monitoring of medicinal products [2].

Regardless of whether the medication error results in harm or not, the detection of medication errors involves similar pharmacovigilance sources such as those employed when identifying adverse drug events (ADEs). Thus, common data sources for medication errors include clinical trials, spontaneous safety reports, observational studies, administrative claims, solicited reports and medical literature [2, 8]. Furthermore, the identification and analysis of medication errors in a similar way as ADEs also rely on the manual review performed by medical/pharmacovigilance assessors [9, 10] and the use of MedDRA® terminology for the coding of drug-related events [3]. Despite the expansion in the MedDRA® terminology to facilitate the coding of medication errors [3], signal detection of medication errors is still a challenge as many of these terms lack sufficient granularity to capture the root cause of the error. This is because medication errors are often a result of a chain of events impacted by human behavior and reasoning. Consequently, pharmacovigilance experts resort to the description of the signal in the narrative to perform the analysis, thus making the process both time consuming and labour intensive. Since the process requires manual review, signal detection of medication errors is further challenged by the previously described increase in submitted reports of medication errors [3].

Investigations of the utility of text mining techniques in the identification of ADEs have increased significantly in recent years and have so far been primarily addressed by academia [11–15] and the FDA [16, 17]. The pharmaceutical industry could potentially also benefit from the use of text mining methods for signal detection. This study explores the possibility of supplementing the manual work performed by pharmacovigilance experts in the identification of medication errors with the use of natural language processing (NLP)-based text mining tools. To achieve this purpose, three methods will be evaluated for their ability to retrieve individual case safety reports (ICSRs) that contain a specific medication error from Novo Nordisk’s (NN) safety database Argus®: the safety surveillance advisor (SSA) method, representing the traditional approach for the detection of a medication error, and two NLP-based text mining methods, namely the I2E text mining and the Center for Protein Research (CPR) text mining. The medication error in question entails the *extraction of insulin from a prefilled pen or cartridge by a syringe* (EIPPCS), which was identified as a safety signal by the EMA in May 2017 [18].

## Methods

The aim of this study is to evaluate the use of two natural language processing text mining methods as complementary tools to the traditional approach followed by pharmacovigilance experts for medication error signal detection by using case narratives from NN Argus Safety® database.

### Data source

The input data for the current study consisted of all ICSRs received by NN in the period from January 1987 to February 2018 and involving all NN insulin products in the form of cartridges, prefilled pen devices and durable pen devices from NN Argus Safety® database. The retrieved 154,209 ICSRs served as the dataset upon which the three methods to extract cases containing the EIPPCS medication error were applied.

### Training dataset

A set of 137 cases served as training data to create and improve the I2E and CPR text mining queries, of which 117 cases were identified in June 2017 by the Safety Surveillance department at NN, using a standard SSA approach, in response to EMA’s assessment of EIPPCS as a safety signal. The search criteria used to identify the 117 cases included solely the fast-acting NN insulin products in a cartridge or prefilled pen presentation over a period ranging from 1 January 2002 to 31 March 2017. Twenty (20) cases were randomly selected from the output of the SSA method and added to the above-mentioned 117 cases. The rationale behind the addition of the 20 cases is to reflect the broader criteria for the data source, i.e. more products included and longer time period, as the input data for the SSA method covered a period ranging from January 1987 to February 2018, and included all NN insulin products in the form of cartridges, prefilled pen devices and durable pen devices. Furthermore, the longer time period also accounts for changes in the description of EIPPCS error in the case narratives, ranging from shorter and less descriptive narratives for older cases to longer and more detailed narratives for the more recent cases.

### Annotated dataset

**Fig. 1:**
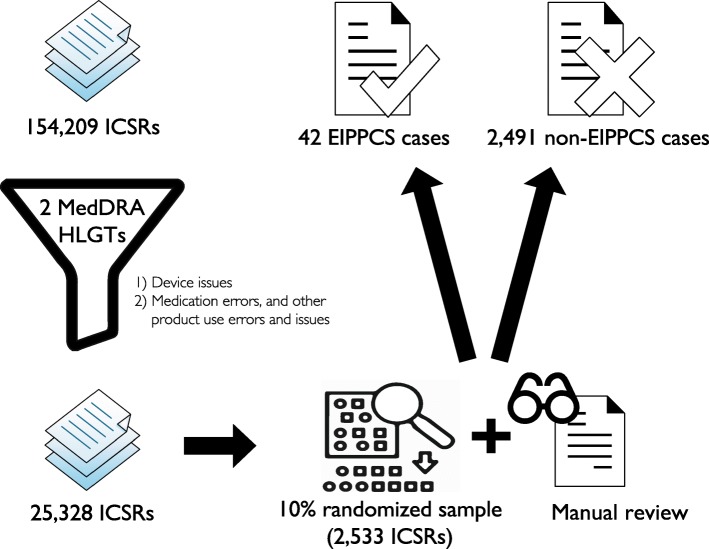
Annotated dataset. Two MedDRA® HLGTs *Device issues* and *Medication errors, and other product use errors and issues* were applied as filters to the starting dataset of 154,209 narratives. This decreased the number of ICSRs to 25,328, and from the latter dataset a 10% random sample corresponding to 2533 ICSRs was taken for manual annotation. MedDRA® Medical Dictionary for Regulatory Activities, HLGTs high level group terms, ICSRs individual case safety reports

The MedDRA® terminology has an internal hierarchy, which permits linking levels of the hierarchy. We collapsed all MedDRA® codes stored in the ICSRs to high level group terms (HLGTs). A dataset for manual annotation was obtained by applying the two MedDRA® HLGTs *Device issues* and *Medication errors, and other product use errors and issues* to filter through the full set of ICSRs meeting the inclusion criteria. A subset of 10% was manually annotated by NKE (Fig. 1). This annotated dataset, which did not include the 117 case narratives, served as the test set to evaluate the performance of the three methods in extracting ICSR narratives that contain the EIPPCS medication error. To account for potential discrepancies in the assessment of EIPPCS between annotators, another annotator SLH reviewed a sample of 100 ICSRs from the annotated set. The inter-annotator agreement was calculated using Cohen’s κ coefficient.

### Safety surveillance advisor method

The SSA method was developed based on learnings from the initially identified 117 cases. For the original signal analysis, case extraction involved the use of two MedDRA® preferred terms (PTs), namely *Wrong technique in product usage process* and *Drug administered in wrong device* as filters to identify relevant cases. Based on information gathered from the narratives of the 117 cases, root causes of EIPPCS error were elucidated as being related to issues with the device itself, the technique in using device or to situations where patients intentionally used the device incorrectly. Examples of phrasing in the narratives that helped elucidate the possible root causes of EIPPCS include: *“It was reported that the patient had been using Levemir® PenFill® and NovoRapid® PenFill® for the last 2 years. It was reported that Levemir® and NovoRapid® was intentionally mixed in the one syringe (to minimise injections in paediatrics patients)”*; “*A patient experienced a pen with air inside and a bubble. She verified this problem as insulin was not coming out of the pen. For this reason, the consumer is taking insulin with syringe.”* and “*A man reported that he mistakenly withdrew insulin out of the NovoLog® FlexPen® with a regular syringe and then injected it back into the FlexPen®*”.

The root cause analysis assisted in expanding the PTs that could potentially be used to code this error from two PTs to the 12 PTs listed in Table 1. The 12 PTs were applied as second filter in the SSA method. The first filter consisted of using the word ‘syringe’ to search across case narratives, as EIPPCS error implies the use of a syringe.

**Table 1:** Twelve MedDRA® preferred terms used as second filter in the SSA method

1. Device failure	
2. Device malfunction	
3. Device use error	
4. Device use issue	
5. Drug administered in wrong device	
6. Drug administration error	
7. Intentional device misuse	
8. Intentional product misuse	
9. Product use issue	
10. Wrong device used	
11. Wrong technique in device usage process	
12. Wrong technique in product usage process	

### Text mining methods

In this project we decided to include two rule-based text mining methods, one method developed for commercial settings and one method currently developed for academic settings. We included both of these to investigate two rule-based text mining methods originating from independent settings. The commercial product included was I2E (Linguamatics, Cambridge, UK), a product already used by the pharmaceutical industry. This is an NLP text mining software that can be applied to various data formats and data sources. It also features an interactive and user-friendly interface. The other text mining method was developed at the Novo Nordisk Foundation Center for Protein Research, University of Copenhagen, and is referred to in this article as “CPR text mining”. This method has a strong focus on the ability to fine tune and processing speed. It also provides a wide range of features that may be independently optimised.

The I2E text mining method and the CPR text mining method both followed an NLP rule-based approach to identify relevant ICSRs. A dictionary referred to as the ‘EIPPCS dictionary’ was created by compiling a list of words and short phrases descriptive of this medication error that were extracted from the 117 case narratives. This dictionary was employed in creating the first set of queries for both text mining methods. Fine tuning of the two text mining methods was performed independently.

#### I2E text mining method

**Fig. 2:**
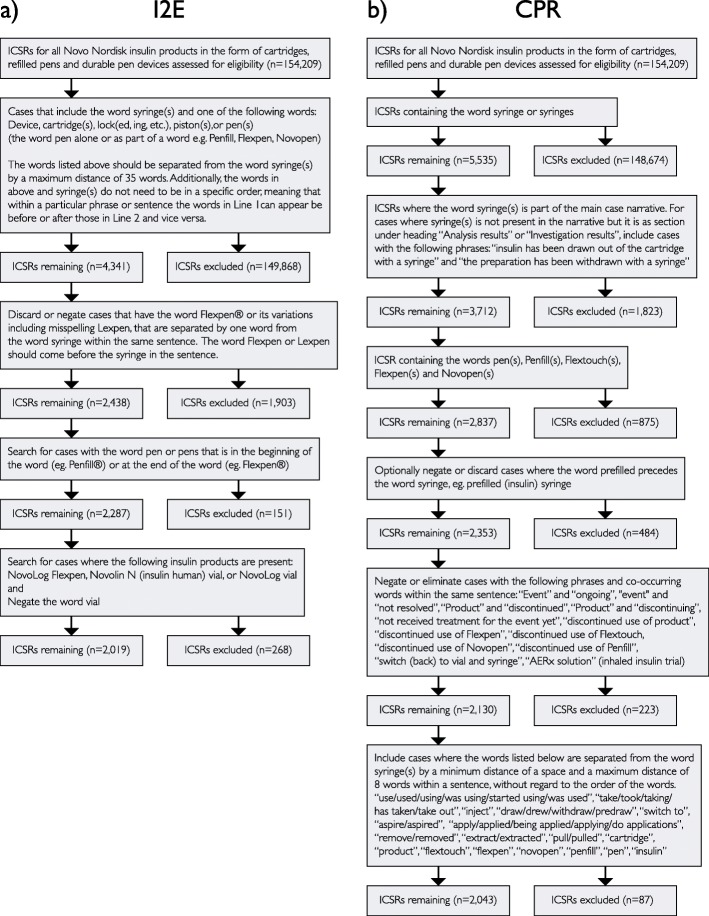
Flow diagrams depicting the I2E method (**a**) and the CPR method (**b**)

The methodology employed for this study was previously described by Milward et al., 2005 [19]. The I2E query relied on simple linguistic pattern extraction rules using a data-driven methodology on the case narrative, to include or exclude cases as presented in the flow diagram (Fig. 2a).

#### CPR text mining method

CPR text mining is based on a named entity recognition (NER) tagger, which identifies matches in input texts. The method was originally designed to identify drug-related adverse events in Danish medical records [14]. However, since the framework of the method is neither context nor language specific, it was possible to apply it for the purpose of this study by constructing a context-specific dictionary that included all insulin pen products marketed by NN, which was used in combination with the EIPPCS dictionary. All of the information extraction was done case insensitive.

The pre-processing steps included isolating the relevant sections from the case narratives for further analysis. This included only analysing the sections associated with consumer mishandling of products and excluding sections concerning subsequent physical and chemical analyses.

After tagging relevant terms, negative filters were applied to disqualified cases. This included cases that contained text indicating that the consumer had not performed the sought drug administration error or that the information originated from an inhaled insulin clinical trial. Finally, we required the word syringe to be equal or less than 8 words separated from words acting as positive filters, as well as these being within the same sentence (Fig. 2b).

### Evaluation measures

#### EIPPCS identification methods

Recall (sensitivity) and precision (positive predictive value) are the parameters used in this study to evaluate the performance of the three methods in retrieving EIPPCS cases. Recall is defined as the proportion of EIPPCS case narratives annotated in the test dataset that were also retrieved by the method under evaluation, among all the EIPPCS narratives listed in the test dataset. Precision is the proportion of EIPPCS narratives retrieved by the method that were also part of the test dataset, among all the EIPPCS narratives retrieved by the method in question.

## Results

We retrieved 154,209 ICSRs fulfilling the inclusion criteria from the NN Argus Safety® database. Applying the two MedDRA® HLGTs to build our annotated dataset resulted in 25,328 ICSRs. Therefore, the random sample consisting of 10% of these was 2533 ICSRs (Fig. 1).

By extracting only ICSRs containing the word ‘syringe’, 5535 ICSRs were identified using the SSA method. This was further reduced by applying the 12 MedDRA® codes, resulting in an end result of 1104 ICSRs. The I2E method identified 2019 ICSRs and the CPR method identified 2042 ICSRs (Fig. 2).

### Annotated dataset

From the reviewed 2533 narratives, 42 were assessed as containing EIPPCS and 2491 as non-EIPPCS case narratives (Fig. 2). The sample of 100 ICSRs that was independently reviewed included the above-mentioned 42 EIPPCS cases and additional 58 cases randomly chosen from the 2491 remaining narratives. The calculated Cohen’s κ coefficient for inter-annotator agreement was 0.96.

### Comparison of method performance

The CPR text mining identified 38 out of 42 EIPPCS cases, the SSA method identified 37 out of the 42 cases and the I2E text mining identified 33 out of the 42 cases.

Recall ranged from 90.4 to 78.5%, with the CPR text mining showing the highest recall (90.4%), followed by the SSA method (88.1%) and the I2E with the lowest recall (78.5%).

**Fig. 3:**
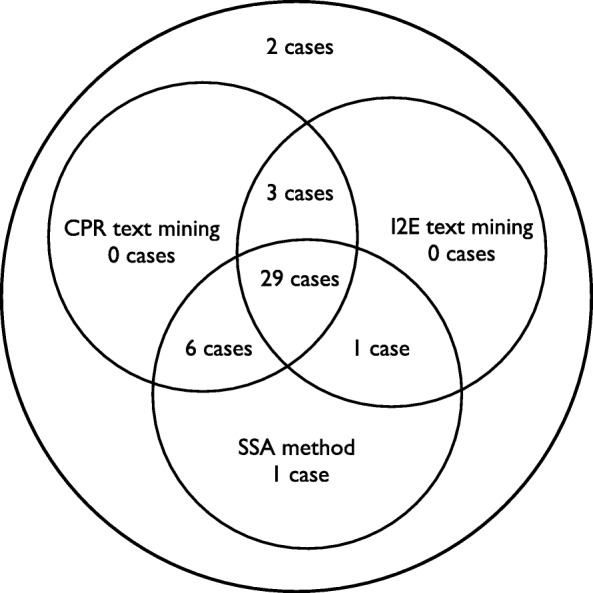
Venn diagram of the true positive cases captured by each method from the annotated dataset. Two TP cases were not captured by any of the methods

The distribution of true positive (TP) cases captured by the three different methods is depicted in Fig. 3. The TP cases captured by the different methods were compared against each other, and the possible root cause for the missed cases was assessed (Table 2). Two cases were missed by I2E text mining due to the context in which the EIPPCS error was described in the narratives. These cases included situations where the product (cartridge) subject to EIPPCS was not clearly stated in the case narrative; but deducible from the product forms. Rule 1 of the I2E text mining was identified as the reason for which two cases were not captured due to insufficient variations of the vocabulary. To overcome this, rule 1 could be adjusted by including more synonyms such as the verbs, e.g. *inject*, *administer* or *use*, which when present in the same sentence as the word *syringe* can be descriptive of the error, and thus capture the EIPPCS cases. Similarly, adjusting the CPR text mining rule 5 to include the verb *administer* would have captured the case that was otherwise retrieved by the SSA and I2E text mining methods. CPR missed one case because it failed to capture the case based on what is written in rule 2, i.e. insulin was withdrawn with a syringe but that was only mentioned in a separate section of the narrative “analysis results” (dedicated section to describe analysis of returned products).

**Table 2:** Number of true positive cases missed by each method and the root cause for missing cases

Affected method	Cause	Number of cases
SSA	PT term not part of the 12 listed in Table 1	3
I2E	Context	2
I2E	I2E rule 4 needs adjustment	2
I2E	Flextouch® not part of the query	2
CPR	CPR rule 5 needs adjustment	1
I2E and CPR	CPR rule 2 was not followed I2E rule 4 needs adjustment	1

**Fig. 4:**
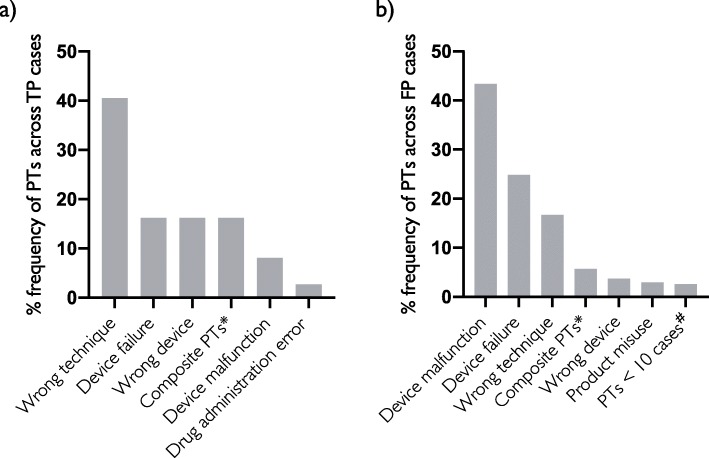
Frequency of PTs across true positive cases (**a**) and false positive cases (**b**). *Composite PTs: Cases in which more than one of the 12 PTs captured the case. #PTs: Several PTs captured less than 10 cases; these have been grouped together in “PTs < 10”. **a** Only 5 PTs were employed to capture true positives in the test set. **b** All 12 PTs were employed to capture false positives; however, the majority captured less than 10 cases

Furthermore, the TP cases identified by the SSA method were analysed based on the frequency of the PTs for the SSA method. The PT *Wrong technique in product usage process* retrieved 40% of TP cases (Fig. 4a).

The number of false negative (FN) cases was 4, 5 and 9 for CPR text mining, SSA and I2E text mining methods, respectively. Two of the FN cases were not captured by all three methods due to the absence of the word “syringe” from the narrative.

Precision was low for all methods with the lowest precision at 1.6% for I2E text mining, 1.9% for CPR text mining and 3.4% for the SSA method. The false positive (FP) cases of all three methods were analysed to investigate if there is a pattern among the cases that could explain the high ratio of FP. The FP cases captured by the SSA method were mapped to the PT term or terms that captured them. *Device malfunction* was identified as the PT capturing the largest number of FP cases, followed by *Device failure* responsible for approximately one-fourth of the FPs cases (Fig. 4b). For the two text mining methods, a comparative post-hoc analysis was made to investigate if there were any concepts present only in the FP data pool and not in the TP data pool, and thus suggesting a potential future filtering step. We identified the most frequent concepts for each text mining method (Table 3). Most of the terms presented are common terms used in daily language but could as well have been used to improve performance in our test set. Thus, filtering based on these terms would be expected to impact precision. Terms related to *“prefilled”* in connection with e.g. “*insulin* “or “*flexpen*” is only represented in the I2E FP data pool. The CPR text mining method already includes a filter to remove the word “*prefilled*” associated with “*syringe*” (Fig. 2b).

**Table 3:** The 25 most frequent terms only found in the false positive dataset and not in the true positive for each of the two text mining methods. If present in both methods, the counts are sorted on the frequencies in the I2E method, otherwise sorted for each method. Terms not present in dataset are marked with '-'

Term	I2E	CPR
insulin syringes	718	516
woman	700	622
elevated	624	567
changes	613	492
prefilled	608	-
prefilled insulin	573	-
flexpen prefilled	545	-
elevated blood	545	495
elevated blood glucose	542	492
the woman	542	471
flexpen prefilled insulin	539	-
introduced	505	404
introduced to	500	397
was introduced	498	401
switched	497	533
was introduced to	494	395
prefilled insulin syringes	491	-
elevated blood glucose levels	489	447
who was introduced	488	399
who was introduced to	486	395
activity	463	-
flexpen prefilled insulin syringes	460	-
conventional	459	657
air	455	414
prior	439	390
while using	-	401
conventional insulin	-	389
man	-	385
prior to	-	375
a woman	-	369
woman reported	-	351
his blood	-	346

## Discussion

Based on the complexity of EIPPCS both in terms of the numerous ways in which the error can be phrased in the narratives, and the lack of MedDRA® PTs that can specifically code such error, this research aimed to evaluate the utility of applying two text mining methods to complement the work performed by pharmacovigilance experts. The main finding is that the two text mining methods I2E and CPR have a similar level of recall and precision as compared to the traditional SSA method.

### Recall

Recall was high for all three methods. The CPR method was most successful in capturing EPICCS cases, with 32 cases in common with the I2E method and 35 cases in common with the SSA method. Analysis of the discrepancies between the methods showed that a recurrent reason was insufficient variations of the vocabulary.

There was a low occurrence of EIPPCS cases in the annotated test and despite annotating 2533 cases only 42 EIPPCS cases were identified. The size of the true cases in the test set could substantially impact the achieved recall, as with a relatively small set of true cases few or even only one case could to a large extent negatively influence the recall of the methods.

The SSA method relied on 12 PTs to capture EIPPCS cases. The 12 PTs were identified based on the analysis of various ways to describe the signal which often relied on a root cause and was thus highly context dependent. For complex signals like the EIPPCS, this is labour-intensive work as no single, or even few PTs, sufficiently describe the signal. Consequently, 12 PTs were selected to capture EIPPCS cases which resulted in a slightly higher precision as compared to the text mining methods. However, despite the slightly higher precision, the SSA failed to capture 3 cases in which the EIPPCS error was coded with PTs not part of the 12 PTs listed in the method. Including PTs to ensure that these 3 EIPPCS cases would be captured would have increased the number of FPs even further. The missed cases are not considered to be due to insufficient coding; MedDRA® coding of ICSRs is a specialist task, which requires specific training and experience. Rather, a correct coding of ICSRs containing medication errors often relies on decoding the root cause of the error, and since human behaviour is the source of the root cause this inevitably makes MedDRA® coding of medication errors a challenging task.

### Precision

Precision for all three methods was at a similar level and low, which can be attributed to different factors. First, the occurrence of the EIPPCS signal is rare as only 1.7% (42 out of 2533) of the test dataset was identified as EIPPCS cases. Reducing the number of false positives identified from scarce events is complicated without unintentionally decreasing the number of true positive cases, as one parameter often influences the other. Second, the EIPPCS signal is characterised by its many fold root causes, meaning the actual signal description varies both in terms of the several root cause scenarios to be described, and the multiple ways a particular root cause can be described. ICSRs are reported worldwide and translated into English from various other languages which can impact both quality and variability of the case narrative. The source of the ICSR may further add to the variability, e.g., a spontaneous case reported by a patient in layman language or a solicited case report from a clinical trial reported by a medical doctor in long and scientifically correct sentences. Thus, the correct identification of an EIPPCS case relies on the correct interpretation of the context. Therefore, improved interpretation of the context could potentially increase precision.

### Rule-based text mining in the pharmaceutical industry

Rule-based approaches were chosen in this project. This is due to the desirable features of such an approach in the pharmaceutical industry, which are not always fulfilled by machine learning methods.

The most obvious reason is the ability to check every single step of a query and that all modifications to the algorithm behave in a predictable and transparent manner.

The ability to check every step is particularly valuable for the identification of medication error signals, where the error is often a result of a chain of events complicated by variations of human behaviour and reasoning and language complexity as detailed above.

Reproducibility and transparency are essential requirements from the authorities to the pharmaceutical industry and it is therefore necessary to avoid any black boxes. Machine learning is based on probabilistic results, where processes are hidden from the human observer, potentially resulting in an unpredictable model. Small pieces of information may significantly change the model and further increase the unpredictability of the model. The more parameters a machine learning approach can compute may result in an improved model but probably at the expense of the human understanding of the model.

Further, rules in a rule-based approach may be produced in a flexible manner, for example extending searches with lexical synonyms from a database. A rule-based system does not need a massive training corpus, which is especially important in this project since we were searching for a very sparse event. Producing a satisfactory corpus to train a machine learning approach would be extremely time consuming due to the sheer size of the task.

There are, however, also obvious advantages of machine learning that we were not able to benefit from. This includes that the method can “learn” from any data, and therefore may produce results soon after a signal has been identified, and such a methodology may be both time and resource saving for detection of less complex signals as compared to rule-based approaches. Here it is also important to point out that poor input data most likely will produce poor results. In the end, the largest disadvantage of the rule-based method is probably the requirement of skilled operators that can construct rules and enhance these rules over time.

Thus, machine learning models seem to lack the flexibility to capture the dynamic nature of safety signal detection and the transparency to meet regulatory requirements.

### Method comparison

Both text mining methods relied on information from case narratives based on which a set of pre-specified rules were generated. The definition of these rules allowed for a more nuanced vocabulary to describe the signal. Although both definition of rules and the pre-processing steps are based on manual work, it is still less labour intensive than the SSA method. In text mining, ideally a balance between precision and recall should be achieved. Nevertheless, given that EIPPCS is a rare phenomenon accentuating recall over precision is often the norm [20]. Thus, identification of a rare signal or insurance of high degree of certainty that most ICSRs are captured, i.e. high sensitivity, happens at the expense of a low precision. We also favoured high recall, since the false positive cases could be further processed by applying filters such as the concepts listed in Table 3, which would only improve precision without at all decreasing recall. Furthermore, the pharmaceutical industry must meet regulatory requirements to report signals [5], and thus prioritising a high recall is required.

Both with the traditional SSA method and the two text mining methods, a large number of FP EIPPCS cases were captured. To explore if there were any apparent pattern which could be suggestive of the means to optimise the methods, analysis of patterns of TP and FP was undertaken. For the SSA method, the PT terms *Device malfunction* and *Device failure* when combined are responsible for capturing over to two-third of the FP cases. Therefore, omitting these PT terms may improve precision although at the sacrifice of TP cases as 5 cases were captured by either of the two PTs. A number of PTs captured zero cases in the test set although these were identified as appropriate PTs in the initial signal analysis investigating the root causes. Thus, the choice of which PTs to include is a delicate balance between detecting the signal without drowning in noise. The same goes for the text mining methods, where distance between keywords set to a specific maximum can fail to include or exclude cases. For the two text mining methods, an analysis was undertaken to investigate if there were any unique terms present in the FP dataset, and which were not represented in the TP dataset, which could be suggestive of a means to improve precision. Most of the identified terms were common language to describe the signal. For the I2E method terms related to *“prefilled”* in connection with e.g. *“insulin”* or *“flexpen”* is only presented in the I2E FP data pool and could therefore be a suggestion for future optimisation. A similar optimisation was already implemented in the CPR method during development.

Leveraging of valuable information for pharmacovigilance purposes from unstructured text in case narratives has been tested by several researchers [15, 21, 22]. Through the use of NLP text mining, Wang et al. [21]was able to uncover novel ADEs, thus drawing attention towards prospective surveillance versus the traditional retrospective approach that is the mainstay of traditional pharmacovigilance. Furthermore, Harpaz et al. [22] demonstrated that combining data from spontaneous reports with data harvested from the narratives of electronic health records through the use of NLP techniques allows for more accurate signal detection.

In this paper, the usefulness of applying text mining as a pharmacovigilance tool is demonstrated for the detection of a rare signal primarily by reducing work load. The rapid growth of safety data to surveillance combined with increasing regulatory requirements call for the development and testing of new methodologies in pharmacovigilance. This study is the first to exploit the use of text mining methodologies on a ‘real world’ safety signal. Surveillance of medication errors is by nature challenging as the cause of error is often driven by human behaviour which is only poorly captured by MedDRA^@^ coding. This means that surveillance of potential medication errors relies on manual and labour-intensive methodology. Our paper provides an alternative to this in the form of a standardised and transparent methodology. The employment of text mining in pharmacovigilance, as described here, need not be limited to the surveillance of potential medication errors but can be used for the ongoing regulatory requests, e.g., obligations in risk management plans and may thus be utilised broadly for signal detection and ongoing surveillance activities.

## Conclusion

This research shows that text mining methods may be useful tools for signal detection as compared to the traditional pharmacovigilance method. In particular, text mining methods may be beneficial when the signal description relies on unstructured text information, e.g. ICSR narrative, in terms of providing a standardised and less labour-intensive alternative to the traditional pharmacovigilance method.

## Data Availability

The datasets generated and/or analysed during the current study are not publicly available due to the content of individual patient safety information but are available from the corresponding author on reasonable request.

## Abbreviations

ADE: Adverse drug event
CPR: Novo Nordisk Foundation Center for Protein Research
EIPPCS: Extraction of Insulin from a Prefilled Pen or Cartridge by a Syringe
EMA: European Medicines Agency
FDA: U.S. Food and Drug Administration
FN: False negative
FP: False positive
HLGT: High level group term
ICSR: Individual case safety report
MedDRA: Medical Dictionary for Regulatory Activities
NLP: Natural language processing
NN: Novo Nordisk
SSA: Safety Surveillance Advisor
TP: True positive
WHO: World Health Organization
